# Three-Year Breeding Cycle of Rainbow Trout (*Oncorhynchus mykiss*) Fed a Plant-Based Diet, Totally Free of Marine Resources: Consequences for Reproduction, Fatty Acid Composition and Progeny Survival

**DOI:** 10.1371/journal.pone.0117609

**Published:** 2015-02-06

**Authors:** Viviana Lazzarotto, Geneviève Corraze, Amandine Leprevost, Edwige Quillet, Mathilde Dupont-Nivet, Françoise Médale

**Affiliations:** 1 INRA, UR 1067 "Nutrition, Métabolisme, Aquaculture", Aquapôle, 64310 Saint-Pée-sur-Nivelle, France; 2 INRA, UMR 1313 "Génétique Animale et Biologie Intégrative", 78352 Jouy-en-Josas, France; Catalan Institute for Water Research (ICRA), SPAIN

## Abstract

Terrestrial plant resources are increasingly used as substitutes for fish meal and fish oil in fish feed in order to reduce the reliance of aquaculture on marine fishery resources. Although many studies have been conducted to assess the effects of such nutritional transition, no whole breeding cycles of fish fed diets free from marine resources has been reported to date. We therefore studied the reproductive performance of trout after a complete cycle of breeding while consuming a diet totally devoid of marine ingredients and thus of n-3 long chain polyunsaturated fatty acids (n-3 LC-PUFAs) that play a major role in the formation of ova. Two groups of female rainbow trout were fed from first feeding either a commercial diet (C, marine and plant ingredients), or a 100% plant-based diet (V, blend of plant proteins and vegetable oils). Livers, viscera, carcasses and ova were sampled at spawning and analyzed for lipids and fatty acids. Although the V-diet was devoid of n-3 LC-PUFAs, significant amounts of EPA and DHA were found in livers and ova, demonstrating efficient bioconversion of linolenic acid and selective orientation towards the ova. Some ova were fertilized to assess the reproductive performance and offspring survival. We observed for the first time that trout fed a 100% plant-based diet over a 3-year breeding cycle were able to produce ova and viable alevins, although the ova were smaller. The survival of offspring from V-fed females was lower (-22%) at first spawning, but not at the second. Our study showed that, in addition to being able to grow on a plant-based diet, rainbow trout reared entirely on such a diet can successfully produce ova in which neo-synthesized n-3 LC-PUFAs are accumulated, leading to viable offspring. However, further adjustment of the feed formula is still needed to optimize reproductive performance.

## Introduction

Aquaculture is currently the fastest growing animal production sector and has been expanding continuously for the last 25 years to meet the increasing demands in terms of fish as food that fisheries cannot meet. Aquaculture now contributes to 40% of the total fish production for human consumption and is expected to reach 60% by 2020 [1,2]. This supposes a growth rate of aquaculture that cannot be achieved by using fish meal (FM) and fish oil (FO) as major dietary sources. Their use in fish production is more than ever limited because of their decreasing availability and high cost. Carnivorous fish species, such as salmonid species, remain among the highest consumers of fish oil (51% of the market share) and the third highest of fish meal (19.5% of the market share) [3]. Among salmonids, rainbow trout (*Oncorhynchus mykiss*) farming represents the main freshwater production in Europe [3], and intense research efforts are being focused on replacing marine feedstuffs (FM and FO) with more easily available plant-based ingredients [4] in their diets.

Replacement of FM by alternative terrestrial plant products has been explored for more than twenty years [4–6]. Although substantial reduction in dietary levels of FM can be achieved, there are still several difficulties to be overcome with regard to total replacement of FM by plant ingredients, even in salmonids. Some previous studies have shown that the complete replacement of FM by plant sources of protein leads to lower growth performance in rainbow trout [5], possibly linked to decreased feed intake [7]. Moreover, poorer digestibility of carbohydrates [8], that are abundant in plant-based diets, and the presence of antinutritional factors [9] can also be responsible for poorer growth performance. With regard to FO, several studies carried out in salmonids (rainbow trout, brown trout-*Salmo trutta*-, Atlantic salmon- *Salmo salar*) have shown that it is possible to replace FO totally by individual or mixtures of vegetable oils, without affecting growth or feed efficiency [10,11], provided that n-3 LC-PUFA requirements are met by the lipids contained in fish meal. A major problem when replacing both FM and FO by plant sources is the lack of n-3 long chain polyunsaturated fatty acids (n-3 LC-PUFAs), such as eicosapentaenoic acid (EPA) and docosahexaenoic acid (DHA). These fatty acids (FA) are important in the fish life-cycle, most notably for their roles in flesh quality [12] and reproduction, egg quality and offspring development [13,14]. In both salmonids and many cultured fish, sexual maturation and gonad development occur at the expense of stored energy, including lipids [15]. Previous studies in rainbow trout have shown that during sexual maturation lipids are initially mobilized from visceral adipose tissue [16], and mobilization from secondary storage sites (e.g. muscle) occurs only in the long term [17]. Moreover, visceral adipose tissue appears to be the primary source of energy for vitellogenesis [18], a crucial step in the female reproductive cycle. After mobilization of adipose fatty acids, this process ensures subsequent hepatic synthesis and export of lipoproteins (vitellogenin) which, together with lipids and vitamins, is finally taken up by the ova through endocytosis, providing energy reserves for ovum and offspring development. During this process, n-3 LC-PUFAs such as EPA and DHA are preferentially incorporated into ova, typically at a ratio of 2:1 [19]. EPA and DHA, as well as n-6 PUFA arachidonic acid (ARA), are recognized as determining factors in egg quality of several species [13,20–25] and in offspring development [26]. In addition to meeting energy requirements of early ontogenesis, one specific role of n-3 LC- PUFAs, in particular DHA, is incorporation into forming membranes and maintaining their fluidity [27]. Salmonids fed a diet lacking in n-3 LC-PUFAs are capable of biosynthesizing DHA via the desaturation and elongation of linolenic acid (available in plant ingredients, such as linseed oil) [17]. However, it has also been demonstrated that such bioconversion is not sufficient to compensate for the lack of dietary n-3 LC-PUFAs, resulting in a significant reduction of these FA in fish tissues [28]. While several studies have examined the effects of a plant-based diet on growth, metabolism and fish flesh quality, the consequences of supplying a plant-based diet devoid of FM and FO across an entire life-cycle on reproductive performance and offspring survival have not been studied to date. This constitutes a significant gap in knowledge, since successful reproduction, egg quality and offspring development are key elements in fish farming [29].

We investigated whether rainbow trout reared on a plant-based diet from first feeding to reproduction were capable of synthesizing sufficient amounts of n-6 and n-3 LC-PUFAs from precursors to survive and reproduce. Furthermore, in addition to reproductive performance the incorporation of n-3 and n-6 PUFAs into ova and the subsequent trans-generational effects on offspring survival were also studied, in order to assess whether female trout exclusively reared on a plant-based diet can produce viable ova and offspring.

## Materials and Methods

### Ethics

The experiment was carried out in strict accordance with EU legal frameworks relating to the protection of animals used for scientific purposes (Directive 2010/63/EU) and guidelines of the French legislation governing the ethical treatment of animals (Decree no. 2001-464, May 29th, 2001). It was approved by the ethics committee of INRA (INRA 2002-36, April 14, 2002). The INRA experimental facility is certified for animal services under the permit number B29-277-02 by the French veterinary services, which is the competent authority and the scientist in charge of the experimentation received training and personal authorization (N°B64 10 003). During the experiment absence or occasional presence of dead (opaque, whitish) or unfertilized (transparent) eggs was daily checked, as well as the eggs shape and size. Dead, unfertilized or not normally shaped/sized eggs were carefully removed with a pipette by competent person in charge of the experiment fish rearing. As well as for the eggs, fish were daily monitored during the study. If any clinical symptoms (i.e. morphological abnormality, restlessness or uncoordinated movements) were observed, fish were sedated by immersion in 2% benzocaine solution and then euthanized by immersion in a 6% benzocaine solution (anesthetic overdose) during 3 minutes, to be certain that death was achieved.

### Diets

The experiment was conducted with two different diets: a commercial (C) diet from “*Le Gouessant”* (Lamballe, France) containing a mix of FM, FO and plant ingredients, and an experimental plant-based diet (V), completely free from FM and FO, which were replaced by a blend of plant ingredients. The latter diet was formulated by UR NuMeA (INRA, Saint- Pée sur Nivelle, France) and manufactured at the INRA experimental facilities (Donzacq, France). Ingredients and compositions of the diets are presented in Table 1. In the C-diet 45% of FM and 50% of FO were replaced by plant ingredients. During the feeding trial, the producer of the commercial diet (*Le Gouessant*) changed the origin of the ingredients (mainly FO), so that fish of the C group received diets (C_1_ and C_2_) with slightly different FA composition over the three breeding years. The V-diet contained only plant protein sources and a blend of vegetable oils (50% rapeseed oil, 30% linseed oil, 20% palm oil). This blend was chosen in order to provide an overall amount of FA classes closely resembling the proportions of FA classes found in fish oil. No n-6 or n-3 long chain poly-unsaturated fatty acids (LC-PUFAs), such as eicosapentaenoic acid (EPA), docosahexaenoic acid (EPA) or arachidonic acid (ARA), were present in the V diet, whereas it contained high levels of 18:3 n-3 (alpha-linolenic acid, ALA) and 18:2 n-6 (linoleic acid, LA) compared to the C diet. The fatty acid composition of the two diets is provided in Table 2.

**Table 1:** Ingredients and composition of diets.

Diets	C	V
Ingredients (g/Kg)		
**Fish meal** *	434	0
Corn gluten	0	170
Soybean meal	163	200
Wheat gluten	0	250
Durum wheat	100	49.8
White lupin	0	57.2
Dehulled peas	86	30
**Fish oil** **	105	0
Soybean oil	105	0
Rapeseed oil	0	62
Linseed oil	0	37
Palm oil	0	24
Soy lecithin	0	20
L-lysine	0	15
L-arginine	0	10
CaHPO4.2H20 (18%P)	0	35
Binder	0	20
Min.-Vit. Premix	7	20
Composition (% DM)		
Crude protein	40	44.8
Crude fat	28	23.3
Energy kJ/g DM	24.5	23.6

*Origin co-fishery products—all species

** Origin co-fishery products—sardines

C: commercial diet *Le Gouessant*, V: experimental 100% plant-based diet

**Table 2:** Fatty acid composition (% of total fatty acids) of diets.

Diets	C_1_	C_2_	V
Fatty acid			
Saturated	23.7	25.8	16.1
MUFA	22.5	38.3	39.8
Σ n-6	8.1	10.0	21.9
18:2 n-6 (LA)	5.7	8.5	21.7
20:2 n-6	0.2	0.4	0.05
20:3 n-6	0.2	0.2	0.0
20:4 n-6 (ARA)	1.3	0.6	0.0
22:2 n-6	0.4	0.1	0.2
22:4 n-6	0.1	nd	0.0
Σ n-3	36.3	20.2	20.1
18:3 n-3 (ALA)	1.5	2.7	20.1
18:4 n-3	2.5	1.9	0.0
20:3 n-3	0.1	0.2	0.0
20:4 n-3	0.9	0.7	0.0
20:5 n-3 (EPA)	17.6	6.9	0.0
22:4 n-3	0.3	nd	0.0
22:5 n-3	1.9	1.1	0.0
22:6 n-3 (DHA)	11.5	6.4	0.0

C _1–2_: commercial diet *Le Gouessant*, V: experimental 100% plant-based diet

C_1_: fed until year-2 spawning; C_2_: fed between year-2 and year-3 spawning

MUFA: monounsaturated fatty acids

*nd*: not detected

### Fish and experimental design

Female rainbow trout were produced at the INRA fish facilities (PEIMA, Sizun, France). Throughout the experiment, fish were reared under natural photoperiod and temperature conditions. Fish were randomly divided into two groups that were reared either on the commercial diet (C) or on the plant-based FM/FO-free diet (V). The dietary treatment was applied from first feeding until the end of the trial. Female fish of the V-diet group were fed twice a day, until apparent satiety. In order to avoid wide differences in body weight at the time of analysis, feed ration of C-group was adjusted to that of V-group, because it is known that feeding rainbow trout with plant-based diets free of marine resources leads to reduced feed intake [5,7], which results in reduced weight gain in large size rainbow trout [30].

At the moment of first spawning (2 years old females), ten females from each dietary treatment were sacrificed by benzocaine overdose, weighed and measured. The ova, liver, digestive tract (intestine with perivisceral adipose tissue) and carcasses (whole gutted fish) were collected from each female and weighed. Three pools of 50 ova from each female were weighed and the average weight (mg) of a single ovum was calculated. Absolute fecundity was measured as number of ova/female and gonadosomatic index (GSI) was calculated as: (gonad weight/total female body weight) x 100.

Rearing of the remaining fish continued with the two dietary treatments (C_2_ and V) up to the next reproduction (3 years old females) when ova were sampled following the same protocol as described for the first.

At each spawning approximately 400 of the collected ova per female were fertilized with a pool of sperm collected from males fed a commercial diet and the survival rate of progeny was measured at the eyed stage, at hatching, and at the swim-up fry stage (before 1^st^ feeding).

All collected tissues, ova and swim-up fry sampled were stored at -80°C until analysis.

### Lipid and fatty acid analysis

Total lipid content of female tissues, ova and swim-up fry was quantified gravimetrically after extraction by dichloromethane/methanol (2:1, v/v), containing 0.01% of butylated hydroxytoluene (BHT) as antioxidant, according to Folch *et al*. [31]. Neutral (NL) and polar lipid (PL) fractions were separated on silica cartridges (Sep-Pak, Waters, Ireland), according to Juaneda and Roquelin [32].

Fatty acid methyl esters (FAME) were prepared by acid-catalyzed transmethylation, using boron trifluoride according to Shantha & Ackman [33]. FAME were then analyzed in a Varian 3900 gas chromatograph equipped with a fused silica DB Wax capillary column (30m x 0.25 mm internal diameter, film thickness 0.25 μm; JW Alltech, France). Injection volume was 1 μl, using helium as carrier gas (1 ml/min). The temperatures of the injector and the flame ionization detector were 260°C and 250°C, respectively. The thermal gradient was as follows: 100–180°C at 8°C/min, 180–220°C at 4°C/ min and a constant temperature of 220°C for 20min. Fatty acids were identified with reference to a known standard mixture (Sigma, St Louis, MO, USA) and peaks were integrated using Varian Star Chromatography Software (Star Software, version 5). The results for individual FA were expressed as percentage of total identified FA methyl esters and as quantities (g/100g tissue) for ARA, EPA and DHA.

### Statistical analysis

Data were analyzed statistically using the R software version 2.14.0 and the Rcmdr package. The normality of distribution and the homogeneity of variance of the variables were tested with Shapiro-Wilk’s and Levene’s test, respectively. When both conditions were satisfied, an independent sample t-test was performed to assess the effects of the dietary treatment; the variables with non-parametric distribution were either normalized with an arcsin transformation or, if the criteria were still not met (some fatty acids), a non-parametric test (paired Wilcoxon test) was used for analysis.

## Results

### Biometric parameters and reproductive performance

Biometric parameters (Table 3) of fish of both groups were measured at the spawning of year-2 and year-3. At the year-2 spawning the body weights of females fed the V-diet were higher (+18%) than that of females fed the C-diet, indicating that limitation of food supplied to the C-fed group may have been too restrictive, but no such significant difference was found at the year-3 spawning. Female body lengths were similar at both spawnings irrespective of the dietary treatment. In terms of reproductive parameters (Table 3), total spawn weight from both C and V-fed females increased at the year-3 spawning, compared to the year-2. At the first spawning, the absolute fecundity (ova/female) of the V-fed females was higher (+17%) than that of the C-fed females. Absolute fecundity was higher at the year-3 spawning than at the year-2 for both the C-fed and V-fed females, and at that time no significant difference was detected between the two groups. Average ovum weight was significantly lower in V-fed fish at both spawnings (year-2: -17%, year-3: -12%). One female among the fish fed the V-diet did not produce any viable ova at the year-2 spawning. The gonadosomatic index of the V-fed females was significantly lower than that of the C-fed females, at both spawnings (-19% year-2 spawning; -28.8% year-3 spawning). A lower gonadosomatic index was observed for both groups at the year-2 spawning compared to the year-3.

**Table 3:** Biometric parameters, reproduction performance and survival rates.

	Year-2 spawning			Year-3 spawning		
Diets	C	V	p-value	C	V	p-value
Biometric parameters						
Fish weight (g)	1185 ± 253	1446 ± 205	<0.05	3453 ± 727	3166 ± 539	ns
Fish length (mm)	395 ± 41	406 ± 20	ns	570 ± 51	558 ± 43	ns
Whole spawn weight (g)	224 ± 45	222 ± 43	ns	495 ± 140	349 ± 84	<0.05
Ova weight (mg)	53 ± 7	44 ± 7	<0.05	65 ± 9	57 ± 6	<0.05
Reproduction and survival performance						
Gonadosomatic index (%)	18.9 ± 2	15.3 ± 1.4	<0.05	14.3 ± 2.3	11.1 ± 2.4	<0.05
Absolute fecundity (ova female^-1^)	4243± 589	5113 ± 994	<0.05	7680 ± 2538	6334 ± 1700	ns
Eyed stage survival (%)	91 ± 4	69 ± 30	<0.05	90 ± 7	84 ± 12	ns
Hatching survival (% of eyed)	90 ± 5	56 ± 29	<0.05	87 ± 10	82 ± 14	ns
Swim-up fry survival (% of hatched)	85 ± 5	50 ± 32	<0.05	84 ± 7	78 ± 13	ns

Data are presented as mean ± SD. *p-values* were produced by independent sample t-test. *ns*: not significant

**Fig 1:**
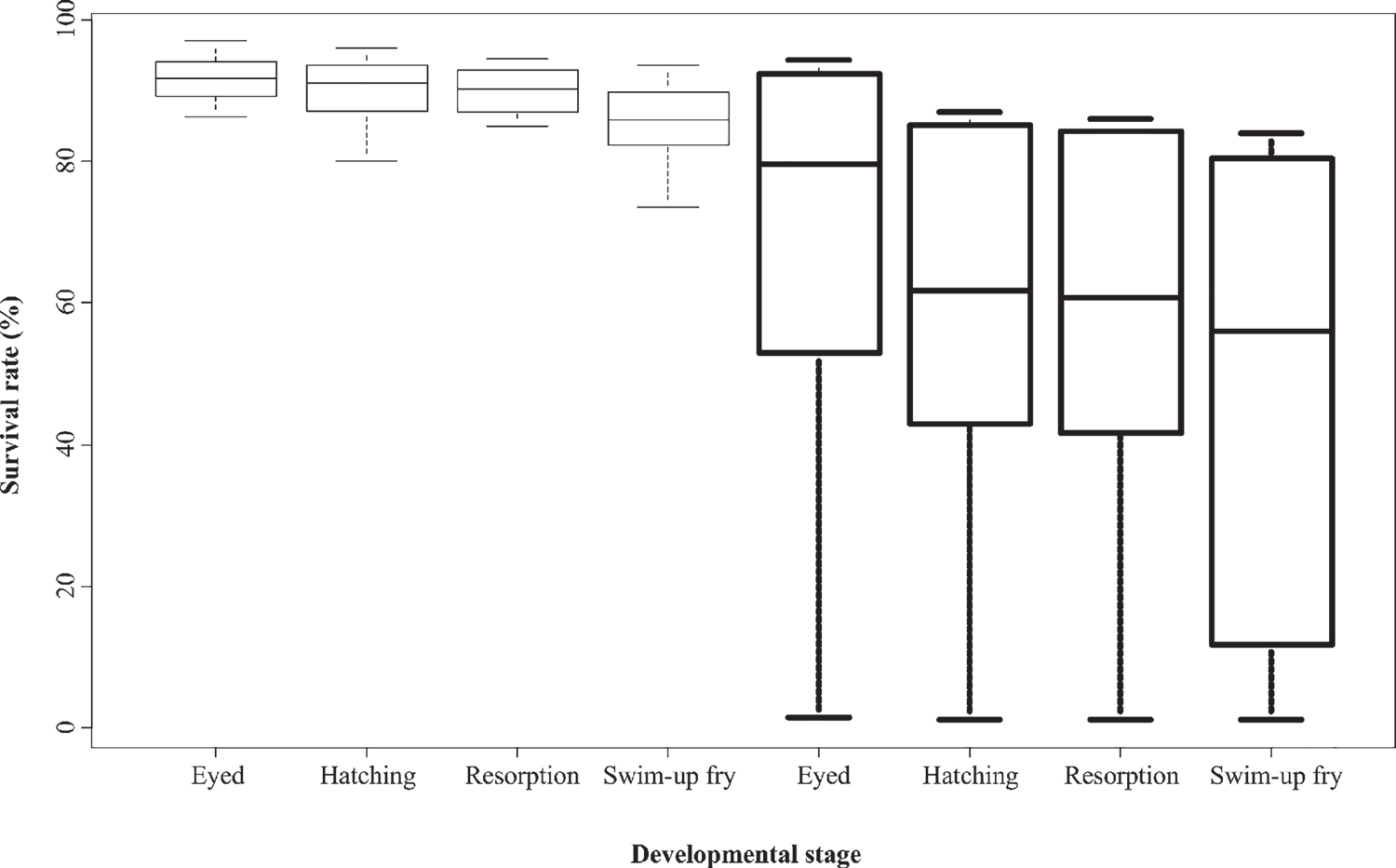
Individual variability of progeny survival rates at different stages from females fed the C and the V diet (in bold).

Rates of progeny survival (Table 3) were assessed as percentage of fertilized ova that reached the eyed stage and that were then able to hatch and to develop into swim-up fry. The survival rates for progeny from the first spawning of V-fed females were lower than for progeny of broodstock fed the C-diet, and wide inter-individual variability was observed for the V-fed group (Fig. 1). However, at the year-3 spawning, no such significant diet-induced differences were detected between alevins from the two treatment groups (Table 3).

### Tissue lipid composition

**Fig 2:**
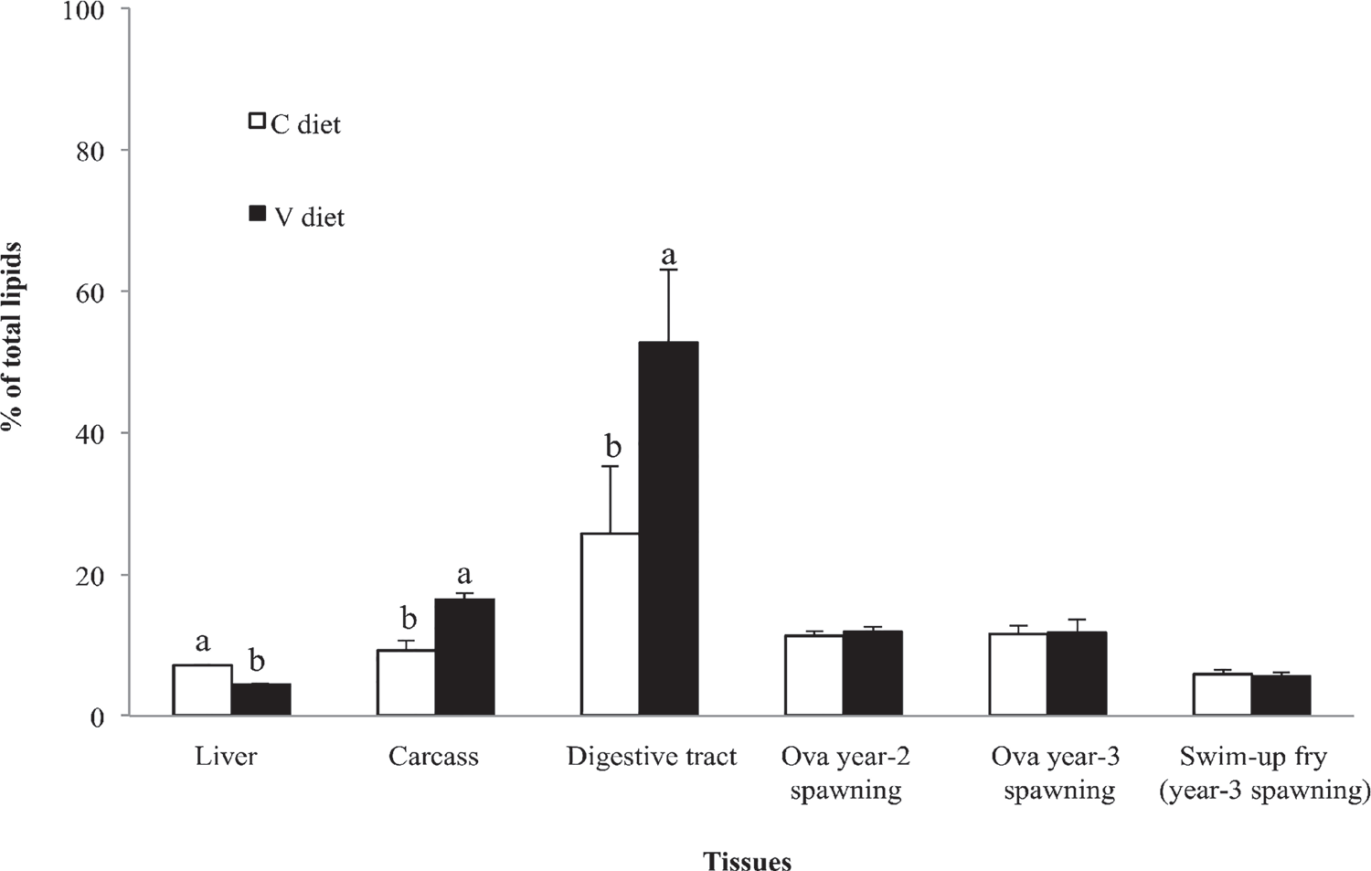
Total lipid content in female tissues, ova and swim-up fry. Data are presented as mean ± SD. p-values were produced by independent sample t-test. Different letters indicates significant differences (*p-value* <0.05).

Fish fed the V-diet exhibited significantly lower lipid content in the liver (-38%), while higher lipid content was found in the digestive tract (+51%) and carcass (+44%) (Fig. 2). The lipid content in the carcass and digestive tract was mainly composed of neutral lipids (NL) for both V- and C-fed groups (>90% and >88%, respectively). However, the lipid content of livers was principally composed of polar lipids (PL) in both V- and C-fed females (65% and 55%, respectively).

In ova from the year-2 and the year-3 spawning (Fig. 2) no significant differences were observed in total lipid content (11%) or in the proportions of NL and PL, which represented 62–65% and 38–35%, respectively, for both female diets.

Similarly, no significant differences were detected in terms of total lipid content (5% i.e. lower than ova), or neutral or polar lipid fractions (68–69% and 31–32%, respectively) in swim-up fry from either treatment group (Fig. 2).

### Fatty acid profile

NL and PL fractions of the tissues collected from sexually mature females, ova from the year-2 and year-3 spawning and alevins were analyzed for fatty acid composition. The proportions of FAs of all the tissues analyzed are presented in Table 4 and Table 5. A significant effect of the dietary treatment was observed for all fatty acids analyzed.

**Table 4:** Fatty acid composition (% of total FA) of livers, carcasses and digestive tracts (polar and neutral lipid fractions).

	Polar lipids			Neutral lipids		
Diets	C	V	p-value	C	V	p-value
Liver						
Saturated	26.6 ± 2.2	21.9 ± 2.4	<0.05	22.1 ± 1.9	14.1 ± 1.5	<0.05
MUFA	13.8 ± 1.4	16.5 ± 2.4	<0.05	23.3 ± 4.0	38.5 ± 5.5	<0.05
n-6 PUFAs	10.8 ± 0.8	20.1 ± 1.0	<0.05	10.6 ± 0.5	21.9 ± 0.6	<0.05
18: 2 n-6	3.0 ± 0.3	8.2 ± 0.9	<0.05	6.4 ± 0.9	16.8 ± 1.3	<0.05
ARA	6.9 ± 0.7	7.4 ± 1.0	ns	3.2 ± 0.7	1.8 ± 0.7	<0.05
n-3 PUFAs	47.1 ± 3.8	40.9 ± 4.7	<0.05	37.9 ± 6.1	23.5 ± 4.7	<0.05
18: 3 n-3	0.5 ± 0.1	3.4 ± 0.9	<0.05	1.5 ± 0.3	8.8 ± 0.8	<0.05
EPA	12.0 ± 1.3	6.1 ± 1.4	<0.05	12.6 ± 2.1	2.7 ± 1.1	<0.05
DHA	29.0 ± 3.8	24.8 ± 3.4	<0.05	16.2 ± 3.7	6.7 ± 3.3	<0.05
Carcass						
Satured	28.3 ± 1.0	23.6 ± 2.0	<0.05	25.1 ± 0.9	15.5 ± 0.8	<0.05
MUFA	15.7 ± 1.7	20.7 ± 2.0	<0.05	30.2 ± 1.6	47.5 ± 0.7	<0.05
n-6 PUFAs	6.2 ± 0.5	21.2 ± 0.8	<0.05	8.5 ± 0.4	21.7 ± 0.4	<0.05
18: 2 n-6	3.1 ± 0.3	14.0 ± 1.3	<0.05	7.1 ± 0.4	18.9 ± 0.3	<0.05
ARA	2.3 ± 0.1	3.9 ± 0.5	<0.05	1.1 ± 0.1	0.3 ± 0.1	<0.05
n-3 PUFAs	45.1 ± 2.8	32.1 ± 2.3	<0.05	31.4 ± 2.1	14.3 ± 0.7	<0.05
18: 3 n-3	1.0 ± 0.1	7.5 ± 0.7	<0.05	1.9 ± 0.1	9.9 ± 0.3	<0.05
EPA	11.9 ± 0.6	6.1 ± 0.8	<0.05	10.1 ± 1.0	0.7 ± 0.1	<0.05
DHA	27.1 ± 2.5	13.9 ± 2.2	<0.05	12.5 ± 0.9	0.9 ± 0.3	<0.05
Digestive tract						
Satured	31.7 ± 1.2	17.9	nc	17.9 ± 1.9	11.7 ± 0.8	<0.05
MUFA	26.8 ± 1.5	35.6	nc	30.9 ± 2.0	48.4 ± 0.9	<0.05
n-6 PUFAs	9.2 ± 0.4	21.4	nc	11.0 ± 0.6	23.0 ± 0.8	<0.05
18: 2 n-6	5.4 ± 0.7	15.2	nc	7.9 ± 0.6	19.7 ± 0.7	<0.05
ARA	2.5 ± 0.5	2.2	nc	1.4 ± 0.2	0.4 ± 0.1	<0.05
n-3 PUFAs	27.1 ± 2.1	22.6	nc	32.9 ± 3.5	15.4 ± 1.0	<0.05
18: 3 n-3	1.1 ± 0.2	6.1	nc	1.6 ± 0.3	8.9 ± 0.6	<0.05
EPA	6.5 ± 1.0	2.4	nc	7.5 ± 0.9	0.6 ± 0.1	<0.05
DHA	14.3 ± 1.7	8.5	nc	13.1 ± 1.7	1.7 ± 0.3	<0.05

Data are presented as mean ± SD; *p-values* were produced by independent sample t-test or equivalent non-parametric test (two-sample Wilcoxon test)

MUFA: monounsaturated fatty acids

*nc*: *p-value* not produced, PL for the V group pooled for practical reasons

**Table 5:** Fatty acid composition (% of total FA) of ova and swim-up fry (polar and neutral lipid fractions).

	Polar lipids			Neutral lipids		
Diets	C	V	p-value	C	V	p-value
Ova (year-2 spawning)						
Saturated	29.5 ± 3.1	28.0 ± 2.9	ns	17.0 ± 2.4	12.9 ± 0.8	<0.05
MUFA	11.3 ± 1.1	14.2 ± 1.1	<0.05	21.7 ± 1.6	34.7 ± 1.6	<0.05
n-6 PUFAs	5.9 ± 0.4	16.6 ± 0.8	<0.05	8.3 ± 0.5	19.9 ± 0.4	<0.05
18: 2 n-6	1.2 ± 0.1	6.0 ± 0.7	<0.05	5.1 ± 0.4	13.1 ± 0.7	<0.05
ARA	3.3 ± 0.2	5.2 ± 0.8	<0.05	1.9 ± 0.1	1.9 ± 0.3	ns
n-3 PUFAs	51.0 ± 3.9	39.6 ± 4.2	<0.05	46.4 ± 3.8	29.6 ± 1.7	<0.05
18: 3 n-3	0.3 ± 0.0	1.8 ± 0.3	<0.05	1.8 ± 0.2	7.7 ± 0.4	<0.05
EPA	12.2 ± 0.8	9.0 ± 1.2	<0.05	17.1 ± 1.7	4.2 ± 0.4	<0.05
DHA	33.3 ± 3.7	22.7 ± 3.8	<0.05	18.2 ± 2.1	9.7 ± 1.4	<0.05
Ova (year-3 spawning)						
Saturated	33.4 ± 2.2	31.3 ± 1.0	<0.05	20.5 ± 1.3	15.1 ± 0.8	<0.05
MUFA	12.2 ± 0.5	15.3 ± 0.7	<0.05	27.1 ± 1.4	39.4 ± 1.1	<0.05
n-6 PUFAs	9.1 ± 0.6	18.1 ± 1.2	<0.05	15.9 ± 0.9	19.7 ± 0.7	<0.05
18: 2 n-6	3.0 ± 0.4	6.2 ± 1.2	<0.05	12.5 ± 0.8	13.8 ± 1.0	<0.05
ARA	3.2 ± 0.1	6.1 ± 1.4	<0.05	1.3 ± 0.1	1.9 ± 0.6	<0.05
n-3 PUFAs	43.3 ± 1.9	33.6 ± 1.8	<0.05	32.1 ± 2.2	23.5 ± 0.8	<0.05
18: 3 n-3	0.4 ± 0.0	1.4 ± 0.3	<0.05	2.5 ± 0.2	7.4 ± 1.0	<0.05
EPA	10.1 ± 0.3	8.7 ± 0.8	<0.05	9.4 ± 1.1	3.4 ± 0.6	<0.05
DHA	29.0 ± 1.4	19.7 ± 1.8	<0.05	14.3 ± 1.4	6.9 ± 1.1	<0.05
Swim-up fry (year-3 spawning)						
Saturated	31.1 ± 2.5	28.3 ± 1.5	ns	18.7 ± 1.2	14.7 ± 0.7	<0.05
MUFA	13.0 ± 0.4	15.1 ± 0.6	<0.05	24.1 ± 0.7	36.4 ± 0.7	<0.05
n-6 PUFAs	8.2 ± 0.4	13.7 ± 0.6	<0.05	15.5 ± 0.2	20.3 ± 0.3	<0.05
18: 2 n-6	3.2 ± 0.1	4.3 ± 0.2	<0.05	11.4 ± 0.3	13.0 ± 0.2	<0.05
ARA	3.3 ± 0.2	6.4 ± 0.6	<0.05	1.7 ± 0.1	2.4 ± 0.2	<0.05
n-3 PUFAs	44.4 ± 2.7	38.6 ± 2.6	<0.05	37.1 ± 1.9	25.9 ± 1.0	<0.05
18: 3 n-3	0.5 ± 0.0	1.3 ± 0.4	<0.05	2.3 ± 0.2	6.6 ± 0.1	<0.05
EPA	9.0 ± 0.7	7.7 ± 0.5	<0.05	9.9 ± 0.6	3.8 ± 0.2	<0.05
DHA	32.5 ± 2.4	28.5 ± 2.8	ns	18.3 ± 1.1	9.6 ± 0.7	<0.05

Data are presented as mean ± SD. *p-values* were produced by independent sample t-test or equivalent non-parametric test (two-sample Wilcoxon test)

MUFA: monounsaturated fatty acids

*ns*: not significant

Saturated fatty acids (SAT) were mainly present in the PL fraction in all maternal tissues and in ova and swim-up fry, with lower values for the V-fed group (Table 4 and 5). In ova from the year-2 spawning and swim-up fry, no significant difference was detected in the PL fraction in terms of SAT content (Table 5), whereas percentages of SAT of the NL fraction were lower in the V-fed group than in the C-fed group (-24% and -21% in ova and swim-up fry, respectively), as well as in all the others tissues analyzed (Table 4).

With regard to monounsaturated fatty acid (MUFA) content, feeding the V-diet led to higher percentages of these FA in all tissues analyzed (+40%), in ova (+31–38%) and swim-up fry (+34%) in both the PL and NL fractions, with a more pronounced effect in the NL fraction. MUFAs were the most abundant FA class in the NL fraction of all maternal tissues, and ova and swim-up fry from V-fed fish.

Total n-6 PUFAs were higher in maternal tissues, and in ova and swim-up fry from the V-fed group (+10–15%). Linoleic acid (LA, 18:2 n-6) was mostly recovered in the NL fraction in all the tissues analyzed from both C- and V-fed groups, with significantly higher values when fed the V-diet. A higher proportion of both n-6 PUFA and LA was found in ova from C-fed females at the year-3 compared to the year-2, both in PL and NL fractions.

ARA was mainly present in the PL fraction of all tissues, and in ova and swim-up fry, irrespective of diet, with higher percentages observed for the V-fed group, except in the digestive tract. Lower percentages of ARA were found in the NL fraction of all maternal tissues with the V-diet (Table 4). Similar ARA content (g/100g tissue) was found in the carcasses and digestive tracts of females from the two groups (Table 6), as well as in the ova at the year-3 spawning (Table 7). Significantly higher (or equal) percentages of ARA were detected in both NL and PL lipid fractions in ova and swim-up fry of the V-fed group compared to the C-fed group (Table 5).

**Table 6:** ARA, EPA and DHA content (g/100g tissue) in carcasses and digestive tracts of C- and V-fed females.

	Diets		
	C	V	*p-value*
*Carcass*			
ARA	0.12 ± 0.03	0.11 ± 0.03	*ns*
EPA	1.05 ± 0.3	0.22 ± 0.1	*<0.05*
DHA	1.44 ± 0.4	0.31 ± 0.1	*<0.05*
*Digestive tract*			
ARA	0.01 ± 0.01	0.02 ± 0.01	*ns*
EPA	0.06 ± 0.03	0.03 ± 0.01	*<0.05*
DHA	0.10 ± 0.05	0.07 ± 0.03	*<0.05*

Data are presented as mean ± SD. *p-values* were produced by independent sample t-test

*ns*: not significant

**Table 7:** ARA, EPA and DHA content (g/100g ova) in ova from second- and third-year spawning.

	Year-2 spawning			Year-3 spawning		
Diets	C	V	p-value	C	V	p-value
Fatty acid						
ARA	0.6 ± 0.1	0.8 ± 0.2	<0.05	1.2 ± 0.2	1.3 ± 0.5	ns
EPA	3.9 ± 1.1	1.6 ± 0.3	<0.05	5.8 ± 1.4	2.1 ± 0.5	<0.05
DHA	6.1 ± 1.0	3.8 ± 0.8	<0.05	6.2 ± 1.8	2.7 ± 0.7	<0.05

Data are presented as mean ±SD. *p-values* were produced by independent sample t-test

*ns*: not significant

Percentages of n-3 PUFAs were higher in the PL fraction compared to the NL fraction. It was the most highly represented FA class in maternal tissues (muscle, liver), ova and swim-up fry. Lower percentages of n-3 PUFAs were observed in all tissues for the V- fed group (-5 to -15%). Higher proportions of linolenic acid (ALA, 18:3 n-3) were detected in the V-fed group, particularly in the NL fraction. Percentages of EPA (20:5 n-3) and DHA (22:6 n-3) were lower in all maternal tissues, ova and swim-up fry from V-fed females (Table 5). Lower levels of EPA and DHA (g/100g tissue) were found in the digestive tracts, carcasses and ova of females fed the V-diet compared to those of the C-fed females (Table 6 and Table 7). In swim-up fry, the DHA content of the PL fraction was in the same range, irrespective of the broodstock dietary treatment (Table 5).

## Discussion

The results of this study prove, for the first time to our knowledge, that rainbow trout can achieve a 3-year breeding cycle including two spawnings events, despite being fed a plant-based diet totally free from marine resources. Our study focused on characterization of the dietary effects on the lipid content and fatty acid profile of female tissues, ova and progeny across two spawning events, because these parameters are known to be key determinants of reproductive success and progeny survival [17, 19].

Several studies have been carried out to investigate the possibility of using plant-based diets in rearing fish but very few of them challenged fish with diets in which both FM and FO were substituted [34]. The use of plant-based diets in aquaculture-raised fish species has revealed limitations, such as reduction in feed-intake [35–38]. The concomitant replacement of dietary FM and FO is known to result in lower growth performance [5,39], probably linked to a combination of lower feed intake and lower feed efficiency [5]. This poorer growth effect of a totally plant-based diet is believed to be mainly related to the replacement of FM and not to the FO substitution in rainbow trout [40,41], European seabass [42] and gilthead sea bream [43]. Pereira *et al*. observed that the replacement of FM by plant proteins both reduced feed intake and resulted in poorer reproductive performance in rainbow trout [7], but it was difficult to be certain that the reproductive performance was impaired by FM replacement or by the reduced feed intake. In the present study we adjusted the feed intake of the C-group to that of the V-group, in order to avoid differences in growth and the potential subsequent effects on spawn [44]. Although at the first spawning this feed restriction was slightly too high, we reached our goal at the spawning of the year after.

### V-diet induces perivisceral and carcass lipid deposition

In teleost fish, stored fat supplies energy in periods of low feed availability and, more specifically, in periods of particularly high energy demand, such as reproduction and smoltification [17]. Depending on the species, fish are able to accumulate fat in different tissues [45,46], e.g. the liver in Atlantic cod [38] and perivisceral adipose tissue and flesh in Atlantic salmon and rainbow trout [5,38]. In the present study, females from both groups had significantly higher lipid content in the digestive tract (Fig. 2) than in the carcass and liver. This was is in accordance with the fact that perivisceral adipose tissue is the main lipid storage site in rainbow trout [47], while the liver does not function as a fat store, despite its important role in FA metabolism [48].

The lipid content in the digestive tract was significantly higher in trout fed the V-diet than in those fed the C-diet, mainly due to the NL fraction. This result confirmed results from previous nutritional studies carried out on salmonids [5,38], thus indicating that feeding a 100% plant-based diet leads to greater lipid deposition in perivisceral fat. Previous studies also demonstrated that maternal dietary lipids and adipose tissue lipids mobilized during previtellogenic and vitellogenic development are transported to oocytes via the circulatory system [27,49,50]. Interestingly, in the present study the total lipid content of ova was similar in both dietary groups (11%) at both spawnings and, furthermore, no significant difference in lipid content was found between swim-up fry (5%) originating from the two dietary treatments. The decrease in terms of total lipid content (from 11% in ova to 5% in swim-up fry) is due to the fact that prior to exogenous feeding the embryo utilizes nutrients derived from the ovum, including lipids [51]. In conclusion, the fat content of ova and fry was not related to the stored maternal body fat.

### V-diet affects FA profile in maternal tissues, ova and offspring

One of the main problems of the concomitant replacement of FM and FO by plant sources in fish feed is modification of the fatty acid (FA) profile, that results in decrease in n-3 PUFAs in the fish body [52–54]. Indeed, vegetable oils do not contain EPA and DHA, and the FA profile of fish tissue is known to mirror the FA composition of the diet [36,38]. Our results were consistent with these previous findings; we observed higher concentrations of MUFA, linolenic acid and n-6 PUFA in NL and PL fractions of all maternal fish tissues investigated, reflecting their abundance in the V-diet. In particular, feeding fish the latter diet led to similar or even higher concentrations of ARA in the PL fraction of maternal tissues, although the dietary intake of ARA was zero. When calculating the quantities of ARA (g ARA/100g tissue) we found higher (or equal) values for the V-fed group in the carcasses, digestive tracts and ova from both spawnings. These results can be explained by the higher proportion of n-6 PUFA in the V-diet (22%) compared to the C-diet (8% and 10%), mainly due to the high linoleic acid content, the ARA precursor. On the other hand, the levels of EPA and DHA, a characteristic component of dietary FO, were lower in all tissues of fish fed the V-diet, as were the concentrations of saturated FAs. The bioconversion of 18:2 n-6 into ARA in the V-fed group seems therefore to be greater than the bioconversion of 18:3 n-3 into EPA and DHA. However, measurable concentrations of both EPA and DHA were found in NL and PL fractions of all maternal tissues of trout reared on the V-diet that was devoid of these FAs. Several previous studies reported that the synthesis of n-3 LC-PUFAs, such as EPA and DHA, as measured by the desaturation and elongation of linolenic acid 18:3n-3, was increased in salmonids fed diets in which dietary FO was replaced by vegetable oils [55–57], although the replacement of FO by vegetable oils resulted in reduced levels of EPA irrespective of the species [36]. The present study is the first in which rainbow trout were fed across the whole life cycle (3 years) without dietary intake of EPA and DHA (concomitant replacement of FM and FO). The fact that non-negligible quantities of these two FA were found, indicated that female rainbow trout are capable of synthesizing EPA and DHA from dietary linolenic acid and to store these two fatty acids in both NL (reserve) and PL (membrane) fractions. However, the EPA and DHA content of tissues, ova and swim-up fry was significantly lower in V-fed trout than in those fed the C-diet (Table 4 and 5), meaning that the biosynthesis of EPA and DHA was not enough to overcome the total absence of these two FAs in the diet.

The degree of modification of the FA composition as a result of dietary FO replacement was significantly different between lipid storage sites (adipose tissue, muscle) and other tissues. Irrespective of the diet, EPA and DHA were mainly stored in the PL fraction of all maternal tissues, confirming selective retention of n-3 PUFAs, particularly DHA, in the membranes [58,59].

Broodstock nutrition is known to play a major role in quality of eggs and larvae, and lipid and fatty acid composition has in particular been identified as the main factor that determines successful reproduction and survival of offspring [60]. Moreover, the lipids derived directly from the dietary intake of broodstock in the period preceding gonadogenesis determine the essential fatty acids vital for early survival and development of newly hatched progeny [27].

With regard to n-6 PUFAs in ova, higher or equal concentrations of ARA were found in NL and PL fractions of ova and alevins from V-fed trout and C-fed trout, suggesting that neo-synthesized ARA from the 18:2 n-6 precursor (LA) is stored in ova. This is important, since ARA plays a major role in the reproductive process [13,61]. Moreover ARA is considered to be the major precursor of eicosanoids in fish [62] and eicosanoids play an important role in ovulation and are probably involved in embryogenesis, hatching and early larval development [63,64].

Our study also revealed increased percentages of n-3 LC-PUFA, EPA and DHA in ova compared to maternal tissues in both C-fed and V-fed fish, suggesting active maternal transfer of these neosynthesized FAs from linolenic acid to the ova. The amounts of EPA and DHA found per 100g of tissue strongly suggested preferential mobilization from perivisceral lipid reserves, rather than from the carcass. As mentioned above, perivisceral fat storage was increased in females fed the V-diet so that sufficient stores were available for the vitellogenic process. The amounts of EPA and DHA found in ova supported the preferential incorporation of these FAs in ova.

EPA and DHA were stored in the reserves (NL fraction) in ova and swim-up fry in addition to the membranes (PL fraction), which are the main sites of deposition. Increased levels of EPA and DHA have previously been described in fish eggs [60,61], supporting the important role that DHA plays in egg and subsequent offspring development. n-3 PUFAs are stored in the reserves for potential use during development [17] or to be catabolized for energy after hatching [65]. For example, DHA is mainly incorporated into the phospholipid-rich vitellogenin which is synthesized in the liver, then transferred via the serum to the developing ova [27]. The accumulation of DHA in the PL fraction of ova has a major biological role as structural phospholipids [27] assuring the fluidity of cell membranes. Our results also showed a greater increase in DHA content from ova to alevins for both groups. This indicates that rainbow trout alevins are able to retain DHA selectively, mainly in the polar lipid fraction.

Measurements of absolute fecundity indicate that the V-diet does not diminish the quantity of ova produced, though ova from V-fed females were smaller. Moreover, with regard to offspring development, feeding the V-diet resulted in a significant decrease (>22%) in survival rate at all stages at first spawning (year-2), confirming previous studies [26,66]. Interestingly, no such significant difference in survival occurred in alevins developing from eggs from the year-3 spawning. It is well known that bigger broodstock produce greater number of eggs and also bigger sized eggs [67]. In our study, comparing the two spawning events, we observed an increase of female weight at the second spawning and a concomitant increase of the ova weight for both females groups. The lower reproductive performance observed for the V-fed group at the first spawning, is possibly linked to the smaller size of eggs, in comparison with those from females fed the C-diet. Indeed, at the year-3 spawning, V-fed females produced ova with a weight comparable with that of ova produced by the C-fed females at the first spawning. We also initially hypothesized that differential accumulation of total lipids or specific changes in the FA profile of ova might be responsible for the differences in survival rate observed between the two spawns of the V-fed females. In this group, ova spawned by 3-years old females had higher ARA (1.3 *vs* 0.8 g/100g ova) and EPA (2.1 *vs* 1.6 g/100g ova) content than those spawned by the 2-years old females. Given the importance of these FAs in reproduction performance [68,69], the higher survival rates observed for offsprings developing from ova spawned by 3-years old females might be linked to the greater ARA and EPA content in ova. However, we also found a lower DHA content, in ova from V-fed females at the year-3 spawning compared to the year-2 spawning. Taking into account the crucial role of DHA in reproduction and embryo development [60], it seems that the higher survival rate observed for offsprings from V-fed females at the year-3 spawning is mostly linked to the egg size and energy reserves than to differences in FA profile of ova.

In conclusion, trout which were reared on plant-based diets completely devoid of long chain PUFAs throughout the entire life cycle (3 years) were able to produce viable ova and progeny. This was probably linked to the fact that the female rainbow trout were remarkably able to synthesize ARA, EPA and DHA from dietary precursors. These neo-synthesized LC-PUFAs were preferentially incorporated into ova, which in fish represent the main sources of nutrients utilized by the embryo prior to exogenous feeding [51]. However, further adjustments of the feed-formula are still needed to optimize reproductive performance, especially at the first spawning.
